# Suicide risk among individuals admitted to an acute psychiatric ward: clinical characteristics, attachment pattern and dignity perception

**DOI:** 10.3389/fpsyt.2026.1871252

**Published:** 2026-07-08

**Authors:** Rosaria Di Lorenzo, Sara Amoretti, Rihab Haddadi, Christal Fogliani, Chiara Manfredi, Martina Morgante, Carolina Bottone, Sergio Rovesti, Paola Ferri

**Affiliations:** 1 Department of Mental Health and Drug Abuse, AUSL-Modena, Modena, Italy; 2 Department of Mental Health and Drug Abuse, Azienda Unità Sanitaria Locale (AUSL)-Reggio Emilia, Reggio Emilia, Italy; 3 Nursing Programme, University of Modena and Reggio Emilia, Modena, Italy; 4 Department of Biomedical, Metabolic and Neural Sciences, University of Modena and Reggio Emilia, Modena, Italy

**Keywords:** attachment style, dignity, psychometric assessment, suicidal behaviour, suicide risk

## Abstract

**Introduction:**

Suicidal behaviour is acomplex phenomenon arising from multiple interactions among different factors.

**Methods:**

This prospective study included 70 individuals hospitalized for suicide risk (SR) between1-1-2024, and 30-9-2025, at the Service for Psychiatric Diagnosis and Care of AUSL-Modena. After Ethics Committee approval, participants completed Columbia-Suicide Severity Rating Scale (C-SSRS), Patient Dignity Inventory (PDI), and Adult Attachment Scale-Revised (AAS-R). Participants’ variables were collected. Data were statistically analyzed.

**Results:**

We underscored the following SR:suicidal ideation(SI) (50%),suicide attempt by ingestion (SA-I) of medications and/or substances (19%), and suicide attempt by violent means (SA-V)(31%).SI involved exclusively adults (p<.001) whereas among minors, SA-Ipredominated (62%, p<.001). SI was significantly associated with living in acquired family (29%) or independently (11%) (p=.000), whereas SA-V with living with origin family (p=.000) and not having children (p=.001). One quarter of sample presented a mood disorder, and one-half a personality disorder. Previous suicide attempts were present in 89% of participants. C-SSRS score was correlated with both PDI and AAS-R.

**Discussion:**

SR was influenced by clinical, psychological, relational, and social vulnerabilities. These findings underscore the need for an integrated clinical approach to meet individual needs with suicidal behaviour.

## 1 Introduction

The theoretical framework and comprehensive understanding of suicidal behavior remain complex due to the wide range of phenotypes that characterize the suicide spectrum (1, 2), from suicidal ideation to death by suicide (3–5). According to World Health Organization, approximately 727,000 persons died by suicide in 2021; suicide was the third leading cause of death among 15-29-year-olds, second for females, third for males. (5). A recent analysis estimated approximately 740,000 suicide deaths per year (6). Furthermore, every minute, four men and six women worldwide require hospital care after a suicide attempt (7). Regarding suicide attempts, WHO (8) estimates that for every death by suicide, approximately 20 people attempt suicide, although this ratio varies depending on countries and the methods used. Although suicide attempt rates are 2–3 times higher in women than in men, suicide mortality is 2.3 times higher among men (9), likely due to more lethal methods and greater difficulty seeking help (5). Hospital suicide is a sentinel event and prevention requires thorough, continuous assessment. Suicide risk peaks in the first days after admission and the first week after discharge (10, 11). Suicide is a complex phenomenon arising from multiple interactions among biological, psychological, social, and environmental factors (12). Contemporary theoretical models (13, 14) suggest that suicide risk develops from a combination of predisposing (distal/diathesis), precipitating (proximal/stress) and mediating factors (2). According to the biopsychosocial model (15), genetic, clinical, psychological, experiential, social, and environmental factors all contribute to suicide risk, although their impact varies widely across individuals.

Distal factors include genetic and familial predisposition (16, 17) and adverse early-life experiences (18–20). Mediating factors partly link distal risk factors to suicidal behavior, enhancing vulnerability such as personality traits, anxiety and aggressive-impulsive traits and cognitive deficits (21–24). Proximal or precipitating factors include prior suicidal behaviors, psychiatric and some organic disorders, psychological factors and attachment style, and socioeconomic, environmental, and contextual variables (25–27).

On the contrary, other factors can counteract the suicide risk. Protective factors may be personal, relational, community-based, or societal. They include coping and problem-solving skills (28), reasons for living (29), family support (30), parenthood (31), social and institutional support (32), and religious involvement (33).

Adams (34) first conceptualized suicidal behavior as an expression of pathological attachment in accordance with Bowlby’s theory (35). Numerous studies confirm the association between insecure attachment and suicidal outcomes (36–38). Anxious attachment is associated with suicidal ideation and attempts, though findings for avoidant attachment are mixed. Psychological mediators include self-criticism and dependency (39). In adolescents, avoidant attachment is a strong predictor of suicide risk, while in older adults’ anxious attachment appears more relevant (40, 41).

Preserving dignity reduces psychological, social, and existential burden and improves treatment outcomes, therapeutic alliance, and adherence (42, 43). Loss of dignity is associated with physical and psychological symptoms such as depression, anxiety, hopelessness, and desire for an early death (44–46). However, research on dignity in people with psychiatric disorders and its relationship with suicide risk remains limited (47).

The aims of this study are to assess the demographic and clinical characteristics, the attachment patterns and dignity perception, according to Chochinov’s conceptualization, of people admitted to an acute psychiatric ward for suicide risk, evaluating the severity of this vital risk and the care necessary to prevent it in hospital.

## 2 Materials and methods

### 2.1 Study design and setting

This observational, prospective study analyzed individuals who were admitted for suicide risk to the Service Psychiatric for Diagnosis and Care (SPDC) of AUSL-Modena in the General Hospital of Baggiovara (Modena) (catchment area of approximately 700,000 residents). In this acute psychiatric ward, 15 beds for voluntary and involuntary admissions (under Law 180/78) and two beds for minors (14–18 years of age) in a separate area are available. The suicide risk (SR) is classified into four levels according to regional guidelines Recommendations for the Prevention of Suicide in Hospital (48): level = 1, no clinical or anamnestic factors for SR; level = 2, mild risk, which indicates the presence of low-intensity SR factors and fleeting suicidal ideation; level =3, moderate risk due to self-harm ideation; level = 4, high risk due to intense suicide ideation and/or recent Suicide Attempt (SA).

### 2.2 Study procedure

We collected all inpatients aged 14 years or older, admitted during the study period from June 1, 2024 to September 30, 2025, with SR level ≥ 2 (48), who agreed to participate in this study, providing their informed consent. We divided SR into the following 3 modality groups in accordance with a previous study in which three distinct SR patterns were underscored in the same setting, corresponding to different demographic and clinical characteristics among inpatients with SR (49): suicidal ideation (SI), suicide attempts (SA) by means drug/substance ingestion, and SA by means violent actions.

### 2.3 Selected variables

For each participant, representative variables relating to demographic, clinical and care aspects were collected, taken from their medical records or from their interview.

- Socio-demographic variables: Age; Sex; Marital status; Parenthood; Housing situation; Education level; Employment status. Clinical variables: Reason for admission; Hospitalization status on admission; Psychiatric diagnoses at discharge (ICD-9-CM); Comorbid medical conditions and substance use; Number of previous suicide attempts; Number of previous psychiatric hospitalizations; Relational/family conflicts, Socioeconomic difficulties.
- Preventive measures implemented in the ward grouped into four domains:

✔ Environmental control and safety (metal detector screening of personal belongings; room inspection; inspection of items brought by relatives/caregivers; monitoring of objects accessible to the patient).
✔ Relational containment (supportive and empathic approach; extended clinical interview with family/caregivers; continuous communication among all healthcare staff).
✔ Behavioral control (personalized monitoring; evaluation of room and bed placement; visual monitoring near the nurses’ station; continuous 1:1 observation when indicated; monitoring for signs suggesting increased suicidal impulse).

• Pharmacological therapy.

### 2.4 Psychometric scales

We assessed our sample within the first five days of hospitalization through the administration of the following psychometric scales:

- The Columbia Suicide Severity Rating Scale (C-SSRS). The scale provides standardized definitions of ideation, behavior, and non-suicidal self-injury, and quantifies their severity across the lifespan and specific time periods (50, 51). The Adult Attachment Scale - Revised (AAS-R). The AAS-R (52, 53) is an 18-item self-report questionnaire rated on a 5-point Likert scale, which measures three dimensions: Closeness (comfort with intimacy); Dependence (perceived ability to rely on others); Anxiety (worry about abandonment or rejection).
- The Patient Dignity Inventory (PDI). The PDI is a self-report scale assessing dignity-related distress developed by Chochinov and colleagues (54), which has been validated cross-culturally, including in Italy (55), and applied beyond oncology, such as in chronic illness and psychiatric settings (43, 56). The PDI consists of 25 items rated on a five-point scale (“no problem” to “an overwhelming problem”), yielding a total score between 25 and 125, evaluating three principal dimensions: Illness-related dignity concerns; Preservation of dignity; Social dignity.

### 2.5 Statistical analysis

Descriptive statistics were computed using:

- Mean ± standard deviation (m ± SD) for continuous variables;
- Percentages and Pearson’s Chi-square test for categorical variables; Standardized Residuals (StR) ≥ 2 were examined to identify cells contributing most to significant associations;
- Multiple linear regression examining associations between C-SSRS scores, as dependent variable, and selected variables, as independent (a stepwise multiple linear regression procedure was performed using backward elimination criteria; variables were removed when p > 0.20);
- Spearman rho’s correlation between C-SSRS, AAS-R, and PDI scores.

A significance level of p <.05 was adopted. Analyses were performed using STATA version 12.

### 2.6 Ethical considerations

The study adhered to Good Clinical Practice guidelines and the Declaration of Helsinki (64th WMA General Assembly, Fortaleza, Brazil, 2013). It was approved by our Institution Review Board which is represented by the Ethics Committee of the Emilia-Northern Area (Prot. AOU 0014348/24, 15/05/2024) and it was authorized by the Modena Local Health Authority (Decision 1403, 29/05/2024). The Ethics Committee later approved an amendment to include participants aged ≥ 14 years (Prot. AOU 0026084/24, 11/09/2024). Written informed consent was obtained from all adult participants and from all parents or legal guardians of minor participants.

## 3 Results

### 3.1 Socio-demographic and clinical characteristics of adult and minor participants

Our sample of adults, who accepted participate in the study and completed psychometric scales, consisted of 55 participants, 18 males (33%) and 37 females (67%), hospitalized for SR during the study period. The mean age was 31.78 (± 14.35 SD) years; mean age was 31 (± 14 SD) years for males and 32.16 (± 14.69 SD) years for females. Overall, 82% of participants were Italians (27% males, 55% females). In our sample, demographic variables did not differ statistically significantly between males and females.

The sample of minor participants, whose parents or legal guards accepted to participate in the study and completed psychometric scales, consisted of 2 males (13%) and 13 females (87%) hospitalized for SR during the study period. Demographic variables did not differ statistically significantly between males and females for any of the parameters examined. The mean age was 15.93 (± 1.10 SD) years: mean age was 15 (± 0 SD) years for males and 16.07 (± 1.11 SD) years for females. Overall, 73% of participants were Italians; 80% had completed lower secondary school, and 93% lived with their family of origin.

### 3.2 Socio-demographic and clinical characteristics associated with SR modality

Analysis of socio-demographic variables associated with the three types of suicide risk, SI (50%), SA by ingestion of medications and/or substances (19%), and SA by violent means (31%), highlighted several significant differences, as reported in **Table 1**. Analysis of clinical variables associated with the three types of SR, allows us to delineate different clinical profiles within the sample regarding family or relational conflicts, socioeconomic difficulties, previous suicide attempts, previous psychiatric treatment and hospitalizations, and clinical reason for hospitalization, as reported in **Table 2**. Some clinically relevant differences in the distribution of psychiatric discharge diagnoses across the three types of suicide risk were found (**Table 3**), although no statistically significant differences were observed.

**Table 1:** Socio-demographic variables by suicide risk modality.

Socio-demographic variables	Suicidal ideation n = 35	SA by medication/substance ingestion n = 13	SA by violent means n = 22	Statistical Test	*p*-value
Sex, n (%)					
Male	12 (34%)	2 (15%)	6 (27%)	Pearson χ² = 1.68	.430
Female	23 (66%)	11 (85%)	16 (73%)		
Age group, n (%)					
Adults	35 (100%)*	5 (38%)	15 (68%)	Pearson χ² = 23.38	.000
Minors	0	8 (62%)*	7 (32%)		
Nationality, n (%)					
Italian	30 (86%)	10 (77%)	16 (73%)	Pearson χ² = 1.78	.777
Other European	1 (3%)	1 (8%)	2 (9%)		
Non-European	4 (11%)	2 (15%)	4 (18%)		
Living situation, n (%)					
Family of origin	9 (26%)	11 (85%)*	19 (86%)*	Pearson χ² = 33.33	.000
New/acquired family	10 (29%)*	0 (0%)	2 (9%)		
Living alone	11 (11%)*	0 (0%)	1 (5%)		
Protected setting	0 (0%)	1 (8%)*	0 (0%)		
Homeless	1 (3%)	0 (0%)	0 (0%)		
Other	4 (11%)	1 (8%)	0 (0%)		
Education, n (%)					
Lower secondary school	12 (34%)	9 (69%)	13 (59%)	Pearson χ² = 12.37	.015
Upper secondary school	14 (40%)	4 (31%)	9 (41%)		
University degree	9 (26%)*	0 (0%)	0 (0%)		
Employment status, n (%)					
Employed	15 (43%)*	0 (0%)	7 (32%)	Pearson χ² = 8.08	.018
Unemployed	20 (57%)	13 (100%)*	15 (68%)		
Parenthood, n (%)					
Children present	14 (40%)*	0 (0%)	1 (5%)	Pearson χ² = 14.44	.001
No children	21 (60%)	13 (100%)*	21 (95%)*		

SA, suicide attempt. *Cells marked with an asterisk indicate standardized residuals (StR ≥ 2), *p* <.05.

**Table 2:** Clinical variables by suicide risk modality.

Clinical variables	Suicidal ideation n = 35	SA by medication/substance ingestion n = 13	SA by violent means n = 22	Statistical test	*p*-value
Pre-existing factors: relational/family conflict, n (%)					
Present	31 (89%)	12 (92%)	15 (68%)	Pearson χ² = 4.96	.084
Absent	4 (11%)	1 (8%)	7 (32%)		
Pre-existing factors: socioeconomic difficulties, n (%)					
Present	22 (63%)	9 (69%)	8 (36%)	Pearson χ² = 5.02	.081
Absent	13 (37%)	4 (31%)	14 (64%)		
Previous suicide attempts, n (%)					
Present	34 (97%)	11 (85%)	17 (77%)	Pearson χ² = 5.51	.063
Absent	1 (3%)	2 (15%)	5 (23%)		
Previous psychiatric treatment, n (%)					
Present	27 (77%)	11 (85%)	15 (68%)	Pearson χ² = 1.28	.528
Absent	8 (23%)	2 (15%)	7 (32%)		
Previous psychiatric hospitalizations, n (%)					
Present	19 (54%)	7 (54%)	7 (32%)	Pearson χ² = 3.02	.220
Absent	16 (46%)	6 (46%)	15 (68%)		
Clinical reason for hospitalization, n (%)					
Agitation	1 (3%)	0 (0%)	0 (0%)	Pearson χ² = 11.78	.300
Socio-environmental emergency	1 (3%)	0 (0%)	0 (0%)		
Hetero-directed aggression	0 (0%)	0 (0%)	3 (14%)		
Depressive symptoms	1 (3%)	0 (0%)	0 (0%)		
Acute psychotic crisis	2 (6%)	0 (0%)	0 (0%)		
Self-harm risk or suicide attempt	30 (86%)	13 (100%)	19 (86%)		

SA, suicide attempt.

**Table 3:** Psychiatric diagnoses by suicide risk modality.

Psychiatric diagnoses	Suicidal ideation n = 35	SA by medication/substance ingestion n = 13	SA by violent means n = 22	Statistical test	*p*-value
Psychiatric diagnosis at discharge					
Schizophrenia spectrum disorder	7 (20%)	0 (0%)	1 (5%)	Pearson χ² = 19.96	.335
Mood disorders	10 (29%)	2 (15%)	5 (23%)		
Anxiety, dissociative, and somatoform disorders	2 (6%)	1 (8%)	1 (5%)		
Personality disorders	8 (23%)	4 (31%)	7 (32%)		
Alcohol/substance use	1 (3%)	0 (0%)	1 (5%)		
Acute/depressive reaction	0 (0%)	2 (15%)	4 (18%)		
Intellectual disability	0 (0%)	1 (8%)	1 (5%)		
Dysthymic disorder	6 (17%)	2 (15%)	1 (5%)		
Major depression/bipolar disorder with depressive episode	1 (3%)	0 (0%)	0 (0%)		
Anorexia	0 (0%)	1 (8%)	1 (5%)		
Associated psychiatric diagnosis					
None	18 (51%)	7 (54%)	10 (45%)	Pearson χ² = 6.78	.341
Personality disorder	14 (40%)	4 (31%)	9 (41%)		
Intellectual disability	0 (0%)	2 (15%)	1 (5%)		
Substance use	3 (9%)	0 (0%)	2 (9%)		

SA, suicide attempt.

### 3.3 C-SSRS, AAS-R, and PDI scores

From the analysis of C-SSRS scores (**Table 4**), active suicidal ideation with specific plan and intent was the most frequent condition in the sample, both over the lifetime period (mean = 4.34 ± 2.15) and in the past month (mean = 4.63 ± 2.04). Overall severity of suicidal ideation showed a generally moderate but stable level of risk over time (**Table 4**). The alpha of Cronbach of C-SSRS was 0.88, suggesting good reliability.

**Table 4:** Psychometric scale scores in the sample.

Scales	Description	Mean ± SD
C-SSRS		
Ideation	Lifetime	4.34 ± 2.15
	Past month	4.63 ± 2.04
Severity	Lifetime	2.29 ± 1.58
	Past month	2.56 ± 1.77
Suicidal Behavior	Lifetime	2.09 ± 1.59
	Past month	1.91 ± 1.42
Lethality	Most recent attempt	2.53 ± 2.26
	Potentially most lethal attempt	2.66 ± 2.34
	First attempt	2.19 ± 2.00
Actual Suicide Attempts	Lifetime	1.63 ± 1.90
	Past 3 months	1.10 ± 0.85
Interrupted Suicide Attempts	Lifetime	1.00 ± 1.41
	Past 3 months	0.89 ± 0.93
AAS-R		
	Closeness	14.33 ± 7.94
	Dependence	12.39 ± 6.97
	Anxiety	16.33 ± 9.92
PDI		
	Illness-related concerns	34.51 ± 10.60
	Preservation of dignity	30.44 ± 10.88
	Social dignity	8.93 ± 3.54

C-SSRS, Columbia–Suicide Severity Rating Scale; AAS−R, Adult Attachment Scale–Revised; PDI, Patient Dignity Inventory.

The AAS-R (**Table 4**) showed a complex pattern of affective and relational functioning with the mean scores of 14.33 ± 7.94 for Closeness, 12.39 ± 6.97 for Dependence, and 16.33 ± 9.92 for Anxiety. The alpha of Cronbach of AAS-R was 0.79, suggesting acceptable reliability.

The PDI (**Table 4**) showed higher scores in the dimensions of “Illness-related concerns” (m = 34.51 ± 10.60 SD) and “Preservation of dignity” (m = 30.44 ± 10.88 SD) compared to “Social dignity” (m = 8.93 ± 3.54 SD). The alpha of Cronbach of PDI was 0.93, suggesting excellent reliability.

### 3.4 Care and preventive measures for SR

The preventive measures which showed statistically significant differences among the three SR modalities were “alertness to any sign of increased suicidal impulse”, which was activated much more frequently in participants who attempted suicide by violent means (55%), and “1:1 observation” which was significantly more frequent among those who attempted suicide by ingestion of medications (31%) or by violent means (23%) compared to individuals who presented SI (Pearson Chi² = 5.68; p = .05); conversely, among patients with SI, the absence of this measure was significantly more frequent (Pearson Chi² = 8.77; p = .012; StR ≥ 2). Behavioural control was applied in almost all cases across the three types of SR.

### 3.5 Correlations between AAS-R, PDI, and C-SSRS

As reported in **Table 5**, the correlation between AAS-R and PDI showed a significant positive correlation (rho = .41; p = .0006). Further significant associations were highlighted between PDI and some dimensions of C-SSRS, particularly lifetime suicidal ideation (rho = .24; p = .0485). Regarding the relationship between AAS-R and C-SSRS, positive correlations were underscored between attachment dimensions and suicidal behaviour: the anxious attachment correlated significantly with lifetime (rho = .28; p = .0241) and the past month suicidal behaviour (rho = .29; p = .0197).

**Table 5:** Correlations between AAS-R, C-SSRS, and PDI.

Variables	PDI	AAS-R
PDI	–	rho = .41, *p* = .0006
AAS-R	rho = .41, *p* = .0006	–
C-SSRS – Suicidal ideation (lifetime)	rho = .24, *p* = .0485	ns
C-SSRS – Suicidal ideation (past month)	rho = .23, *p* = .0560	ns
C-SSRS – Suicidal behavior (lifetime)	ns	rho = .28, *p* = .0241
C-SSRS – Suicidal behavior (past month)	ns	rho = .29, *p* = .0197

### 3.6 Multivariate linear regression

In **Table 6**, the statistically significant results of multivariate linear regression models, estimated using backward stepwise procedure, aimed at identifying demographic and clinical factors that significantly influenced C-SSRS scores as the dependent variables, are reported.

**Table 6:** Multivariate linear regression: variables statistically significantly associated with C-SSRS scores (dependent variable).

C-SSRS scores	Predictor	Coefficient	*p*-value; 95% CI
Suicidal ideation – lifetime	Absence of previous psychiatric treatment	-1.39	*p* = .020; 95% CI: -2.54 to -.23
Severity of Suicidal ideation – lifetime	Age	.03	*p* = .048; 95% CI:.00 to.05
Suicidal ideation – past months	Reason for admission: self-harm risk or suicide attempt	6.00	*p* = .030; 95% CI: 2.07 to 9.94
	Reason for admission: socio-environmental emergency	6.71	*p* = .014; 95% CI: 1.43 to 12.01
	Absence of previous hospitalizations	-1.39	*p* = .008; 95% CI: -2.40 to -.38
Severity of Suicidal ideation – past months	Absence of previous hospitalizations	-.89	*p* = .031; 95% CI: -1.69 to -.081
	Absence of comorbidity	-1.17	*p* = .020; 95% CI: -2.15 to -.19
Suicidal behavior – lifetime	Previous suicide attempts	1.33	*p* = .019; 95% CI:.23 to 2.44
	Absence of previous hospitalizations	-.93	*p* = .013; 95% CI: -1.65 to -.20
Suicidal behavior – past month	Personality disorders	.74	*p* = .028; 95% CI:.08 to 1.40
	Alcohol/substance use	1.53	*p* = .018; 95% CI:.27 to 2.79
	Previous suicide attempts	1.45	*p* = .005; 95% CI:.46 to 2.45
Severity of most recent suicide attempt	Protected facility	6.45	*p* = .008; 95% CI: 1.75 to 11.14
	Absence of socioeconomic difficulties	1.13	*p* = .049; 95% CI:.00 to 2.26
	Protected facility	6.21	*p* = .011; 95% CI: 1.47 to 10.96
	Absence of socioeconomic difficulties	1.31	*p* = .025; 95% CI:.17 to 2.45
Severity of first suicide attempt	Absence of previous psychiatric hospitalizations	-1.18	*p* = .018; 95% CI: -2.16 to -.21
	Non-European nationality	1.38	*p* = .048; 95% CI:.01 to 2.74

## 4 Discussion

The results of this study highlight the multidimensionality of SR among people hospitalized in an acute psychiatric ward, in line with literature (5, 57). The predominance of females confirms the well-known “gender paradox” (58, 59), whereby women show more frequent suicide attempts and more frequent contacts with health services than men, maintaining lower lethality than males (9). Mood disorders were present in about one quarter of the sample, and personality disorders were found in half of the sample associated with a primary psychiatric disorder, confirming the frequent association between SR and these psychiatric disorders (60–64). In 74% of cases, hospitalization was voluntary, suggesting good adherence to treatment in case of inpatients with SR, differently from other psychiatric conditions (65).

In our analysis, minors more frequently presented reactive diagnoses or situational emotional states, in line with evidence describing adolescence as a developmental period of vulnerability, in which emotional regulation is not yet consolidated (66–69). Most of the minors in our sample lived with their family of origin, which suggests that SR may be related to conflicting relationships or difficult conditions within the family, as highlighted in the literature (70).

We observed different patterns of SR modality between adults and minors: suicidal ideation was almost exclusively represented among adults, whereas ingestion of medications or substances predominated among minors. This pattern is attributable both to the typical impulsivity and acute emotional conflicts and the limited availability of highly lethal means in adolescence as highlighted by literature (71).

Housing conditions, education level, employment status, and parenthood also appear to influence self-harming behaviours. We reported that SI was associated with living with a new/acquired family or living alone as a single person, suggesting complex relational dynamics or, conversely, a condition of loneliness. On the contrary, the majority of inpatients who attempted suicide by ingestion of medications/substances or violent means lived with their family of origin, suggesting a possible emotional involvement with family members and caregivers. Other socio-demographic factors differently associated to the suicide modalities were education and employment status: SI was more frequent among inpatients with higher education (university degree) and employment, whereas SA with violent means were more frequent among unemployed adults with low-to-medium education and without children, possibly associated with increased experiences of failure, hopelessness, and isolation. The variable “having children” showed a significant pattern: parenthood appears associated only with ideation and not with suicide attempts, suggesting a possible protective effect of emotional bonds and parental responsibility in preventing the transition to suicide action, in line with literature findings (31). In fact, SA by means of ingestion of medications/substances was more prevalent among adults without children consistent with lower suicidal planning (72).

More lethal suicidal behaviours, such as the use of violent means, were associated with personality disorders and mood disorders, particularly with impulsive and aggressive dimensions and emotional dysregulation, aspects widely documented in suicide research (73).

### 4.1 Suicide risk, attachment, and dignity

Insecure attachment, particularly anxious attachment, was associated with higher scores for both lifetime and recent suicidal behaviour, confirming literature (74) that describes these styles as predictive of ineffective emotional regulation, relational dependence, and greater vulnerability to experiences of abandonment.

Compromised dignity, as reflected in PDI scores, correlated with the intensity of suicidal ideation, a finding consistent with studies that frame “dignity distress” as a mediator between experiences of failure, loss of personal value, and persistent suicidal ideation (75).

According to our correlation results among the three scales, we found that both lifetime and recent suicidal ideation were positively and significantly correlated with PDI scores, whereas lifetime and recent suicidal behaviour on C-SSRS was positively and significantly correlated with AAS-R scores. This suggests two different psychological profiles for suicidal ideation and suicidal behaviour, consistent with the distinctions made by the C-SSRS. These results, although limited by the cross-sectional and exploratory nature of these analyses, lead us to hypothesize that suicidal ideation may be exacerbated by feelings of failure related to one’s personal dignity, whereas anxious and avoidant attachment may be associated with self-harming actions aimed at avoiding or escaping particularly stressful situations.

### 4.2 Analysis of suicidal risk predictors

In our analysis, the absence of previous psychiatric contacts represents one of the most consistent protective factors, indirectly suggesting that greater vulnerability is observed in individuals already known to psychiatric services. Previous suicide attempts represent a strong predisposing factor, confirming the robustness of this predictor in international literature (76). Similarly, the presence of personality disorders and substance use is associated with a significant increase in recent suicidal behaviours, probably due to impulsivity, emotional dysregulation, and difficulty managing stressors.

Other relevant elements concern the care context: living in a protected facility is associated with greater severity of the most recent attempts and of the potentially most lethal attempts, indicating that these variables are linked to more fragile clinical conditions. The absence of economic difficulties, though seemingly counterintuitive, also emerges as a predisposing factor for the severity of SR, suggesting that serious suicidal behaviour is not necessarily tied to social difficulties but may stem from inner suffering that is not always explained by socioeconomic precariousness. As reported in the literature (77), more advanced age is associated with greater lifetime severity of ideation (78). Overall, our regression model highlights that SR increases particularly in individuals with pre-existing vulnerability in their clinical history and in those with complex psychiatric disorders, as indicated by literature (79).

### 4.3 Care for suicide risk

Analysis of preventive and care measures for SR in a psychiatric ward showed that clinical and nursing interventions were modulated differently according to the severity and type of risk, confirming literature that suggests a stratified, personalized, and multidimensional approach for the management of suicidal behaviour (76, 80, 81). In our sample, staff were alerted at every sign of increasing suicidal impulse in case of SA by violent means (83%). The highest proportion of high-intensity measures such as continuous surveillance (1:1 observation), complete removal of potentially harmful objects, and, in some cases, activation of a “red code” or heightened alert for suicidal impulse was observed among patients who attempted suicide using violent means. This finding is fully consistent with extensive literature that identifies violent methods as markers of higher lethality, greater determination, and an acquired capacity to act on suicidal intent (13, 14). Violent methods in SA more often required intensive care measures, suggesting the importance of the concept of “acquired capability for suicide” described by the interpersonal psychological theory (82, 83), according to which exposure to pain, impulsive and aggressive traits, and previous attempts increase the threshold for fear of death and self-injury.

### 4.4 Limitations and strengths of the study

This study has several limitations. First, the relatively small sample size, particularly for minors, makes it difficult to draw generalizable conclusions or to perform robust comparisons between groups.

Second, the observational, single-centre and cross-sectional design and the absence of a control group limit the generalizability of the findings and reduce internal and external validity. In addition, data collection may be influenced by the quality of clinical documentation, which is not always uniform or complete. The clinical heterogeneity of the sample also represents a potential limitation: patients had very different diagnoses, complex illness histories, and varying levels of severity due to the real-world setting. Another limitation concerns the use of self-report psychometric scales, whose scores may be influenced by emotional state, level of insight, and hospitalization condition.

Finally, the stepwise regression model applied in this study can be subject to criticism for potential overfitting and model instability. Given the exploratory nature of this pilot study and the relatively limited sample size, backward stepwise regression was used as a variable selection procedure. Results should therefore be considered hypothesis-generating and interpreted with caution. Future studies with larger samples are needed to confirm the stability of the identified predictors.

Alongside these limitations, the study has several strengths. It was conducted in a real clinical setting, providing a concrete and faithful description of people hospitalized for SR and of the staff who manage SR in daily practice. All psychometric scales used were translated and validated in Italian and correlation between different psychological dimensions permit us to define a psychological profile of SR not completely highlighted up to now. A comparison between adult and minor suicidal behaviour was carried out, highlighting clinically significant differences. The main findings of this study, although limited generalizability beyond the Italian context, could have transcultural relevance due to its theoretical framework (attachment theory, dignity concept).

## 5 Conclusions

This study analysis highlights that suicidal behavior cannot be interpreted as the result of a single factor, but rather emerges from the interaction between individual vulnerabilities, clinical history, family dynamics, attachment patterns, and subjective perception of dignity, underlining the importance of not neglecting to investigate any personal, familial, environmental and emotional element, as well as the psychopathology of a person with SR.

The study compared adults and minors, showing that suicidal ideation was found exclusively in adults and not among minors. Minors more frequently exhibited suicide or harmful behaviors, such as ingestion of medications or substances, suggesting a pattern attributable to age-related impulsivity, limited access to highly lethal means, and the presence of emotional conflicts and acute reactions rather than structured psychopathological conditions. In adults, by contrast, ideation is often linked to inner experiences of suffering, frequently more structured, such as persistent suicidal ideation, which they are more able to express explicitly than minors probably due to more developed thinking skills.

Analysis of the different suicidal modalities confirmed the presence of distinct patterns. Ideation is associated with greater autonomy and more reflective capacity, while ingestion tends to emerge in contexts of family conflict, emotional instability, and dependence on the family of origin. Attempts with violent means, though less frequent, are concentrated among individuals with complex psychopathological profiles, limited support resources, and long-standing psychological suffering. Housing conditions, education level, employment status, and parenthood also appear to influence self-harming behaviors, indirectly showing how relational and social networks impact SR.

Correlations between AAS-R and PDI scales show that attachment dimensions and personal dignity are particularly indicative of specific SRs. Individuals with anxious–avoidant attachment, prone to fear of abandonment and highly sensitive to judgment, are more exposed to suicidal behavior. At the same time, those who experience a loss of dignity, understood as personal worth and continuity of self, tend to develop more intense suicidal ideation.

The predictive models further confirm an observation long recognized in clinical practice and supported by our sample: previous suicide attempts represent one of the most important risk factors for ongoing SR. Conversely, the absence of previous hospitalizations is associated with lower severity, indirectly confirming that more severe psychiatric conditions requiring hospitalization may result in increased self-harm risk. The presence of personality disorders or substance use significantly increases the likelihood of recent suicidal behaviors.

From a care perspective, our results underline the importance of highly personalized treatment and care, with particular attention to the prevention and management of impulsivity and emotional lability, as well as containment of hopelessness. Surveillance, management of ward spaces, the ability to detect changes in behavior, and the construction of a trusting relationship are indispensable tools for ensuring safety and supporting patients through the most critical phases.

In conclusion, our study shows that SR is a complex phenomenon that can never be reduced to a single element. Effective management requires an integrated approach that combines clinical assessment, attention to subjective and relational experiences, targeted psychological interventions, and personalized clinical care for each individual need.

## Data Availability

The datasets presented in this article are not readily available due to concerns regarding participant/patient anonymity. Requests to access the datasets should be directed to r.dilorenzo@ausl.mo.it.
